# Supplementary material for: Design, Synthesis, and Biological Evaluation of N-Acylhydrazones and Their Activity Against Leishmania amazonensis Promastigotes
Source: Microorganisms. 2025 Jul 2;13(7):1563. doi: 10.3390/microorganisms13071563 (PMC12298860; doi:10.3390/microorganisms13071563)
Supplement: Supplementary file 1 [file microorganisms-13-01563-s001.zip › RMN amostras-20241114T155656Z-001.zip!/RMN amostras/composto 19 -PMM 87-17 13Ca.pdf]

PMM 87-17 – Joao Mafra
Op. Eliane

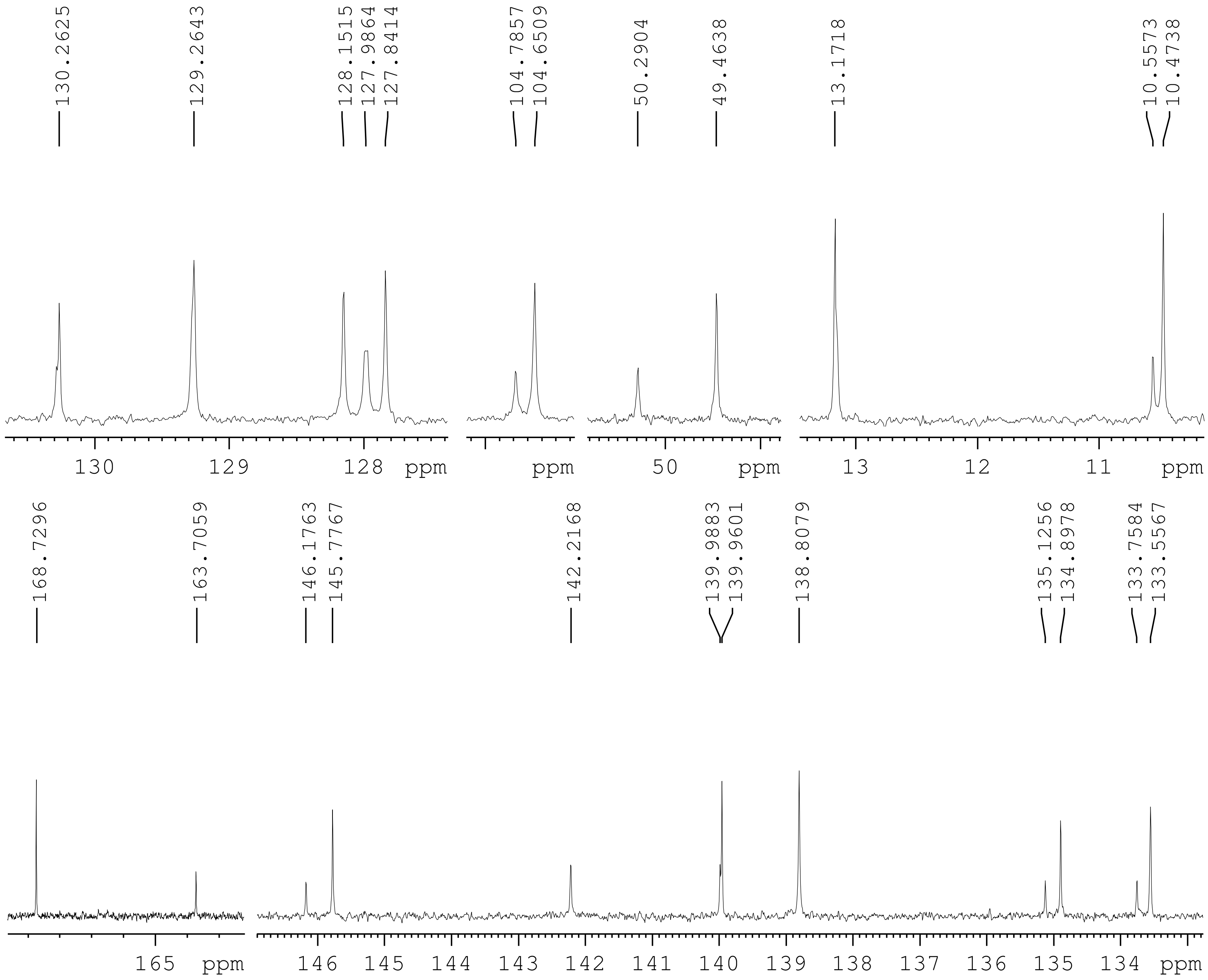

Current Data Parameters

NAME	si103181811
EXPNO	11
PROCNO	1

F2 - Acquisition Parameters

Date_	20180315
Time	11.01 h
INSTRUM	spect
PROBHD	Z122623_0041 (
PULPROG	zgpg30
TD	65356
SOLVENT	DMSO
NS	2048
DS	4
SWH	24038.461 Hz
FIDRES	0.735616 Hz
AQ	1.3594048 sec
RG	46.47
DW	20.800 usec
DE	18.00 usec
TE	298.0 K
D1	1.00000000 sec
D11	0.03000000 sec
TD0	1
SFO1	100.6278593 MHz
NUC1	13C
P1	10.00 usec
PLW1	35.50000000 W
SFO2	400.1516006 MHz
NUC2	1H
CPDPRG[2	waltz16
PCPD2	90.00 usec
PLW2	7.30000019 W
PLW12	0.11919000 W
PLW13	0.05995100 W

F2 - Processing parameters

SI	32768
SF	100.6178560 MHz
WDW	EM
SSB	0
LB	1.00 Hz
GB	0
PC	1.40
